# Exsudats maculaires massifs compliquant des télangiectasies juxtafovéolaires idiopathiques de type 1

**DOI:** 10.11604/pamj.2013.16.47.3063

**Published:** 2013-10-10

**Authors:** Zouheir Hafidi, Rajae Daoudi

**Affiliations:** 1 Université Mohammed V Souissi, Service d'Ophtalmologie A de l'hôpital des spécialités, Centre hospitalier universitaire, Rabat, Maroc

**Keywords:** Exsudats maculaires, télangiectasies, micro-anévrysmes, fond d’œil, macular exudates, telangiectasia, microaneurysms, eyes fundus

## Image en médecine

Nous rapportons un cas de télangiectasies juxtafovéolaires unilatérales de type 1 A révélées par une baisse d'acuité visuelle progressive de l’œil gauche, évoluant depuis 7 mois chez un jeune patient de 25 ans sans antécédents pathologiques notables. L'examen du fond de l'oeil gauche notait un placard exsudatif de la région maculaire entourant des anomalies de la microcirculation paramaculaire temporale. Le reste de l'examen ophtalmologique est par ailleurs sans aucune anomalie visible, avec une périphérie rétinienne normale, une chambre antérieure et vitré clairs sans stigmates d'inflammation. L'examen de l’œil droit est normal. L'angiographie révèle une importante dilatation du lit capillaire péri et parafovéolaire avec plusieurs microanévrysmes et une importante diffusion de la fluorescéine aux temps tardifs. Nous n'avons pas noté de retard de remplissage veineux ni d'anomalies vasculaires de la périphérie rétinienne. Un bilan comprenant une glycémie à jeun, un dosage de l'hémoglobine glyquée, un bilan inflammatoire et une échodoppler des vaisseaux du cou, est revenu négatif. Les telangiectasies rétiniennes juxta fovéolaires représentent un groupe hétérogène de pathologies vasculaires rétiniennes, qui ont en commun une dilatation anormale des capillaires rétiniens juxta fovéolaires, associée à une perméabilité vasculaire accrue pouvant être à l'origine d'exsudations, parfois hémorragiques entrainant une baisse plus ou moins importante de l'acuité visuelle. On distingue plusieurs variantes selon la classification de Gass et Blodi, partageant les patients en 3 groupes. Notre patient rentre dans le groupe 1 type A de cette classification. Selon Gass il s'agirait d'une forme de la maladie de Coats et serait alors congénitale.

**Figure 1:**
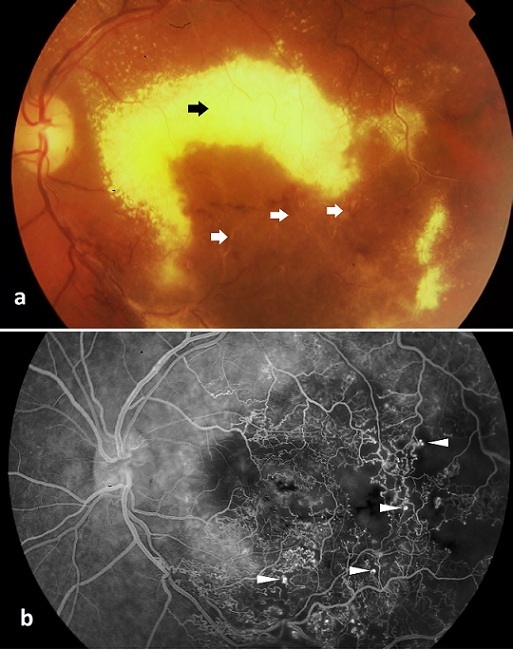
(a) Aspect du fond d’œil gauche montrant un placard exsudatif massif (flèche noire) entourant des anomalies vasculaires juxtafovéolaires (flèche blanche); (b) Angiographie à la fluorescéine au temps intermédiaire montrant une importante dilatation du lit capillaire temporo-maculaire avec des micro-anévrysmes (tête de flèche blanche)

